# Passive and Motivated Perception of Emotional Faces: Qualitative and Quantitative Changes in the Face Processing Network

**DOI:** 10.1371/journal.pone.0040371

**Published:** 2012-06-29

**Authors:** Laurie R. Skelly, Jean Decety

**Affiliations:** 1 Department of Psychology, The University of Chicago, Chicago, Illinois, United States of America; 2 Department of Psychology, Department of Psychiatry and Behavioral Neuroscience, and the Center for Cognitive and Social Neuroscience, The University of Chicago, Chicago, Illinois, United States of America; The University of Melbourne, Australia

## Abstract

Emotionally expressive faces are processed by a distributed network of interacting sub-cortical and cortical brain regions. The components of this network have been identified and described in large part by the stimulus properties to which they are sensitive, but as face processing research matures interest has broadened to also probe dynamic interactions between these regions and top-down influences such as task demand and context. While some research has tested the robustness of affective face processing by restricting available attentional resources, it is not known whether face network processing can be augmented by increased motivation to attend to affective face stimuli. Short videos of people expressing emotions were presented to healthy participants during functional magnetic resonance imaging. Motivation to attend to the videos was manipulated by providing an incentive for improved recall performance. During the motivated condition, there was greater coherence among nodes of the face processing network, more widespread correlation between signal intensity and performance, and selective signal increases in a task-relevant subset of face processing regions, including the posterior superior temporal sulcus and right amygdala. In addition, an unexpected task-related laterality effect was seen in the amygdala. These findings provide strong evidence that motivation augmentsco-activity among nodes of the face processing network and the impact of neural activity on performance. These within-subject effects highlight the necessity to consider motivation when interpreting neural function in special populations, and to further explore the effect of task demands on face processing in healthy brains.

## Introduction

Perceiving and processing emotional facial expressions are natural abilities that pervade the daily life of human beings. The ability to extract information from the expressions of others and make inferences about their mental states is essential to successfully engage in social interactions. Regions of the brain implicated in face and emotion processing, such as the amygdala and fusiform gyrus, are also targeted as regions of interest when investigating individuals with interpersonal deficits, including those with autism and psychopathy.

Visual information from faces is processed via a distributed network. Portions of the inferior occipital gyrus, fusiform gyrus, and superior temporal sulcus form the ‘core’ system for face processing [1]. Identity or unchangeable aspects of the face are processed by the fusiform gyrus [2], while expression, gaze, and other variable aspects of the face are processed by the superior temporal sulcus [3]. Additional processing occurs in the ‘extended’ face processing system: the amygdala and insula process salient emotionalexpression [4]–[6], the inferior frontal gyrus handles semantic information [7], [8], and reward-associated areas such as the orbitofrontal cortex and ventral striatum are thought to process information such as beauty and sexual valuations [9], [10].

When differences in social behavior are attributed to dysfunction in regions that process social and emotional information such as the amygdala and fusiform gyrus, these conclusions implicitly rest on the assumption that responses to emotional and face stimuli are largely automatic, such that in the presence of such stimuli, any difference in activity in the relevant networks is attributable to dysfunction in that network. However, recent lines of research have indicated that within individuals, response characteristics in these regions are likely dependent on other factors. For instance, in autism spectrum disorders, the fusiform gyrus and the amygdala have long been suspected loci of dysfunction contributing to social deficits [11]–[13]. Functional imaging studies have demonstrated atypical BOLD response in the amygdala during the viewing of faces, though the direction of the difference is sometimes higher in controls [14], but sometimes greater in the ASD group [15] or positively correlated with symptom severity [16]. Interestingly, Dalton and colleaguesfound that the amygdala activation was positively correlated with the amount of time that participants fixated on the eyes of the faces, indicating that selective attention may play a role in differences in neural activity during face viewing. In fact, in a study that controlled for attention to the face stimuli, amygdala activation was in fact greater in the ASD group [17]. In the fusiform gyrus, responses to face stimuli were reduced in participants with ASD as compared to controls in several studies [18]–[21]. However, equivalent or increased fusiform activation was seen in ASD participants when the stimuli were faces that were familiar to them, indicating that interest in the stimuli might play a mediating role [22]–[24].

**Figure 1:**
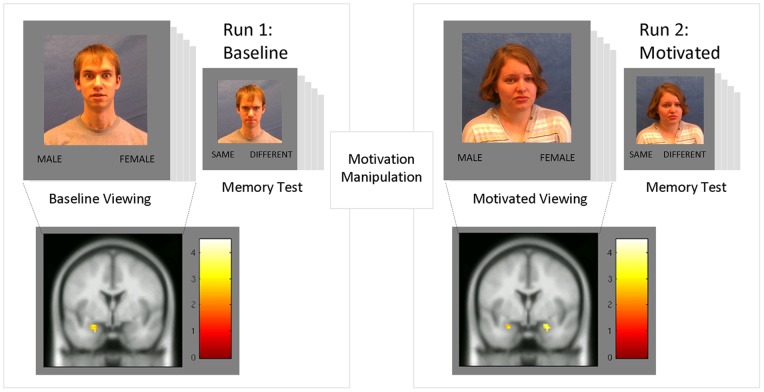
Schematic representation of the task design. Participants viewed dynamic clips of emotionally expressive faces and made gender judgments. After a memory test, they were given feedback on their performance and asked to improve their accuracy and reaction time by ten percent.In Run 1, the man pictured in the Memory Test would be “same” for identity but “different” for emotion. In Run 2, the woman pictured is “same” for identity and emotion. Other probes featured individuals not seen in the baseline viewing block. As a motivational incentive, participants were told that reaching their goal in one try would allow them to get out of the scanner early. Otherwise, they would have to repeat the protocol again in a third run of the task. The brain images below illustrate amygdala activation during the two runs of the task: left-sided only during baseline and bilaterally during motivated viewing.

In psychopathy, as in autism, the amygdala through its connections to the ventromedial prefrontal cortex is a favored putative source of interpersonal and emotional deficits [25]. Again, although results in the anticipated direction have been observed (e.g., [26]), other studies have found either no difference in amygdala activation to emotional stimuli [27], or have seen increased activation in this structure as compared to controls [28]. Interestingly, it has been hypothesized that emotion processing in psychopathy might be the result of broader deficiencies in the allocation of attention [29]. In results echoing those seen in autism, a recent study on adolescent males [30] found that although severity of psychopathic traits predicted poorer performance on a fear recognition task, fixation time on the eyes of the stimulus faces correlated with fear recognition accuracy.

If in healthy individualsneural responsesto emotional faces in these regionswereinvariant and mandatory, theywould be immune to variance in attention or motivation, and would provide a solid basis from which to draw conclusions about face- and emotion-processing circuits in clinical disorders. Alternatively, however, individual differences in interest or motivation to participate in the task could be confounded with the disorder being investigated. Thus the potential for response modulation by factors such as attention or motivation is crucial to the interpretation of between-group differences in these regions.

Neuroimaging evidence supports the notion that certain classes of stimuli, such as faces, which carry a high level of biological salience are processed in an automatic and mandatory way, particularly by the amygdala. For instance, Vuilleumier and colleagues [31] recorded BOLD responses to fearful faces that were either targets or distractors in a matching task and found that amygdala activity was equivalent regardless of the task-relevance of the stimuli (though responses in the fusiform gyrus were reduced when the stimuli were distractors). Similar results were also found when the stimuli were presented foveally [32].

In contrast, a growing body of evidence demonstrates that situational factors such as attentional load can reduce neural responses to these otherwise-privileged stimuli. One study found that by sufficiently increasing the attentional load required by the matching task (drawing attention away from the fear face distractors), amygdala responses to these unattended faces were indeed reduced [33]. Several subsequent studies have replicated this finding of reduced neural activity to unattended faces during heavy attentional load (for a review see [34]).

Beyond attentional load, Righart and de Gelder [35] found changes in N170 responses to fearful faces dependent on the perceptual context: the waveform had greater amplitude when faces were presented superimposed on a fearful scene than on a neutral background. Further, a visual search task has demonstrated modulation of amygdala activity to irrelevant emotional distractors both by attentional set and by trait anxiety [36]. As such, the notion that faces and emotional stimuli are processed by the amygdala in an invariant and completely mandatory way is dubious, and evidence supports that situational and individual factors contribute to the magnitude of the response.

In order to untangle the effects of motivation or interest from differences due exclusively and directly to a disorder of interest, one must first better understand such effects on face- and emotion-sensitive regions in healthy brains. Does increased motivation to attend to and process emotional faces change the magnitude of signal change in the face processing network? It is the hypothesis of this study that increased motivation to actively attend to faces will result in changes not only in response magnitude, but in the relative contributions of different nodes of the network and in the patterns of connectivity exhibited by the amygdala and fusiform gyrus.

Further, changes due to motivation (rather than other factors like practice effect or learning) should be accompanied by changes in regions of ascending modulation of arousal. The locus coeruleus, a subcortical structure found on each side of the rostral pons, is the source of norepinephrine which mediates the functional integration of the whole attentional brain system [37], [38]. This structure is implicated in the contribution of somato-visceral information to higher level processing of affective information via the amygdala and basal forebrain [39]. Selective engagement of this area, and particularly interaction between it and the face processing network, would serve as a reliable check for the motivation manipulation.

The current study investigated effects of the motivation to attend to emotional faces on neural responses and functional connectivity in face- and emotion-processing regions in healthy adults. Short videos of people expressing emotions were presented to volunteers while they were scanned, followed by a surprise memory test of the identity and the emotions of the faces presented. The procedure was then repeated with an incentive to pay greater attention to the stimuli. The participants were told that they would have two chances to improve their performance, and that success on the first try would allow them to end the scanning session early (a potent motivator). During the second run of the task they viewed a new set of emotional expression clips with augmented motivation to attend to the faces and their emotions.

**Figure 2:**
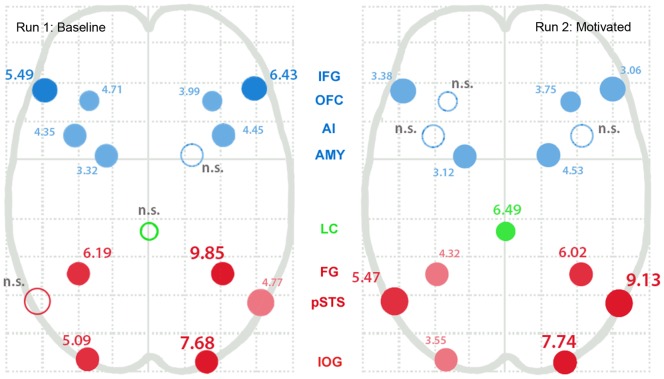
Baseline and motivated BOLD response in nodes of the face processing network. Peak T values for nodes of the face processing network and ascending arousal (locus coeruleus) during encoding of emotional expressions during baseline and motivated viewing. Nodes are labeled via the central column of abbreviations: core network nodes are displayed in red (FG  =  fusiform gyrus, IOG  =  inferior occipital gyrus, pSTS  =  posterior superior temporal sulcus), extended network nodes are displayed in blue (AMY  =  amygdala, AI  =  anterior insula, OFC  =  lateral orbitofrontal cortex, IFG  =  inferior frontal gyrus), and the locus coeruleus (LC) is in green. Saturation and font size indicate significance level (p<0.01, p<0.005, p<0.001, corrected).

**Table 1:** Regions of interest – contrast values and performance correlation.

	LEFT					RIGHT				
	Peak T	x	y	z	r	Peak T	x	y	z	R
Inferior Occipital Gyrus										
Baseline	5.09	−42	−82	−12	0.432 (em)	7.68	40	−72	−12	–
Motivated	3.55	−46	−80	−12	0.439 (em)	7.74	40	−76	−16	
Fusiform Gyrus										
Baseline	6.19	−38	−38	−26	–	9.85	44	−38	−26	0.432 (id)
Motivated	4.32	−40	−34	−26	0.478 (em)	6.02	40	−40	−24	0.502 (em)
Superior Temporal Sulcus										
Baseline	–	–	–	–	–	4.77	56	−38	2	–
Motivated	5.47	−62	−54	12	0.508 (em)	9.13	64	−34	10	0.454 (em)
Amygdala										
Baseline	3.32	−22	−6	−20	–	–	–	–	–	–
Motivated	3.12	−26	0	−22	−0.29 (id)	4.53	24	−2	−22	0.356 (em)
Inferior Frontal Gyrus										
Baseline	5.49	−50	26	26	–	6.53	48	28	18	–
Motivated	3.38	−52	30	20	0.452 (em)	3.06	60	26	14	0.429 (em)
Orbitofrontal Cortex										
Baseline	4.71	−48	38	−6	–	3.99	48	46	−8	0.453 (em)
Motivated	–	−24	36	−12	0.429 (em)	3.75	28	20	−24	0.425 (em)
Anterior Insula										
Baseline	4.35	−40	20	−4	–	4.45	46	20	−6	−0.450 (id)
Motivated	–	–	–	–	–	–	–	–	–	−0.430 (id)
Locus Coeruleus										
Baseline	–	−2	−34	−12	0.422 (id)					
Motivated	6.49	−4	−28	−14	0.450 (id)					

Note: The *r* –value represents the correlation between the peak T value per subject and performance on the memory task, as measured by *D*'. The type of memory score is given in parentheses (id  =  memory for identity; em  =  memory for emotion).

## Methods

Data were acquired from eighteen healthy right-handed volunteers (aged 18–40, nine women), each scanned in a single session after completing structural images and one unrelated task. The participants were free of Axis I or neurological disorders or brain lesions, had never sustained a head injury resulting in loss of consciousness greater than thirty minutes, and had no metal on or inside of the body. All participants were informed of the procedures and risks involved in the study and signed a consent form to participate. The study was approved by the Institutional Review Board at the University of Chicago.

### Task Design

Participants viewed ninety-six videos of facesexpressing happiness, sadness, fear, disgust, anger, and pain, under two motivational conditions in a rapid event-related fMRI design (see Figure 1). The clips (2.2 seconds in duration)were presented using E-Prime2 Professional (Psychology Software Tools, Inc. Pittsburgh, PA) in pseudo-randomized order selected using OptimizeDesign [40], with jittered inter-stimulus intervals of 2.4 to 4.8s(mean 3.6s), totaling 8.5 minutes per run. Functional images were acquired on a 3-Tesla Philips Achieva Quasar scanner at the University of Chicago Brain Research Imaging Center, using a pulse sequence with TR/TE 2200/26, flip angle  = 80°, field of view 230×230 mm, matrix  = 76×76cm, and full-brain coverage in contiguous, interleaved, 4mm slices.

In the baseline condition, participants were asked to make a gender discrimination judgment using their response keypad after the presentation of each video. After twenty-four faces, instructions appeared on the screen announcing a surprise memory task. The participants then viewed a second set of twenty-four faces and indicated whether each person had been seen in the previous trial. For yes responses, it then asked whether the face had previously expressed the same or a different emotion. Each test run of twenty-four faces included ten new individuals and fourteen previously-seen individuals, seven of whom expressed the same emotion and seven who expressed a different emotion.

Between runs, the participants were given feedback about their accuracy and average response time. They were instructed visually and verbally that they needed to improve each metric by ten percent to complete the experiment: if they reached the target after the first run, the scanning session would be completed and they would be allowed out of the scanner. If not, they were told they would have to complete a third run of the task. This incentive was chosen over a monetary reward with an eye to future replications in clinical populations, such as incarcerated inmates, for whom monetary rewards might be prohibited, and because a moderate incentive may avoidundesirable complications such as performance anxiety.

Video clips of actors expressing emotions were generated in the laboratory and edited to duration 2.2 seconds. Clips were selected for quality and realism of expression and validated by members of ourresearch team who were trained in the Facial Action Coding System (A Human Face, Salt Lake City, UT). Each expression category was represented in equal proportions within and between the baseline and motivated conditions.

The motivation manipulation as employed in the current study made it necessary to present the two runs such that the participant received the “baseline” condition before the manipulation was revealed. For this reason it was not possible to counterbalance the two runs. This will be addressed further in the discussion.

### Analysis of Performance

Memory performance for person identity and for facial expression was assessed by calculating four d' scores for each participant. Person identity d' scores were calculated for each run using hit and false alarm rates out of the complete pool of twenty-four trials. Since emotion memory was only probed on those trials in which the participant responded that they had seen the face in the previous run, hits and false alarms were calculated as proportions of those trials in which the participant had correctly reported having seen the face before, ranging from 10–14 trials per subject per run.

### Image Processing and Analysis

The functional images were processed using SPM8 (Wellcome Department of Imaging Neuroscience, London, UK) in Matlab (Mathworks Inc., Sherborn, MA, USA). Volumes were co-registered to the EPI template, realigned and resliced to 2mm cubic voxels, then normalized to MNI space and smoothed with an 8mm Gaussian kernel using DARTEL: individual subjects' structural T1 images were coregistered to the functional images, segmented, and warped to the MNI template. The resulting normalization parameters were then applied to the functional images themselves. Movement parameters from realignment were recorded for use as covariates of no interest in the statistical model. First-level statistics were calculated for each subject in each task by fitting the data to a hemodynamic response function curve including time and dispersion derivatives. Regressors were modeled for each of four categories of face videos(encoding and recall trials in the baseline and motivated conditions), with movement parameters included as covariates of no interest. Contrasts for baseline encoding, motivated encoding, and the subtraction contrast of motivated minus baseline encoding were entered into second-level random effects analyses for groupwise summary.

Correlations with memory performance were calculated at the group level using d' values for actor identity and facial expression memory, respectively, as performance covariates. Results are reported for *a priori* regions of interest at a statistical voxelwise cutoff of p<0.001 with spatial extent threshold k = 25 voxels.

Functional connectivity was conducted using psychophysiological interaction (PPI) for five *a priori* seed regions from each subject: left and right medial fusiform gyrus, left and right amygdala, and the locus coeruleus. PPI is a powerful statistical tool for exploratory analyses of task-related connectivity: for each seed region, a whole-brain general linear model is run which identifies clusters that covary with seed-region activity during the contrast of interest. It does not require *a priori* identification of target regions as in other methods of measuring connectivity, such as dynamic causal modeling (DCM). Each PPI seed eigenvariate was taken from a 3mm-radius spheresurrounding the peak voxel in that region from the participant's collapsed contrast of all encoding trials. Anatomical localization was determined using the MNI template brain atlas and labels in the xjview toolbox for SPM8 (www.alivelearn.net/xjview8/).

A supplementary analysis was conducted to describe amygdala activation to each expression within each motivation condition. Separate regressors for each emotion X condition stimulus type (e.g., Angry_Baseline, Angry_Motivated, Fear_Baseline, Fear_Motivated, etc.) were entered into a first level model, and contrasts for each subject were analyzed at the group level via a random-effects analysis.

## Results

### Behavior

Memory performance was analyzed separately for identity and for emotion matching. Reaction times for both measures were significantly faster in the motivated run (id: baseline 866ms, motivated 564ms, paired t  = 5.63, p = 0.00005; em: baseline 831ms, motivated 573ms, paired t  = 3.20, p = 0.006). Identity memory performance, measured using d', improved significantly in the motivated run (baseline: 2.07, motivated: 2.77, paired t = 2.35, p = 0.03) though memory performance for emotion decreased to a similar extent (baseline: 1.92, motivated: 1.27, paired t = −2.32, p = 0.04). All subjects either volunteered or affirmed that they had felt greater motivation to perform well on the task during the second run of scanning.

### Functional Imaging results

Clusters of robust activation were present for most nodes of the face processing network in both the baseline and motivated runs of the face memory task, though magnitude of response in each condition varied from node to node. Interestingly, a pattern of correlation between face processing network activation and memory performance for emotion was seen across nodes in the motivated condition, but was largely absent in the baseline condition. A schematic diagram displaying peak T-values for each node of the face processing network in the baseline and motivated run is provided in Figure 2, andadditional detailsare included in Table 1.

The core system of the face processing network consists of the inferior occipital gyrus (IOG), the fusiform gyrus (FG), and the posterior superior temporal sulcus (pSTS). Bilateral IOG and FG were robustly and equivalently active in both runs, while pSTS activity increased bilaterally in the motivated run: the left was active only in the motivated condition, while the right was active in both but more so in the motivated run. Correlation between memory performance and the magnitude of face node BOLD response at baseline was significantly correlated across subjects only in the right FG. In the motivated condition, significant correlations were foundbetween memory performance for emotion and all nodes of the core system except for the right IOG.

Nodes of the extended system include the inferior frontal gyrus (IFG), the orbitofrontal cortex (OFC), the anterior insular cortex (AIC), and the amygdala. The IFG and OFC followed the same general response pattern as the IOG and FG: bilateral activation was significant in both runs, but was correlated with emotion memory performance only in the motivated condition, with two exceptions. Left OFC was not significantly activated at the group level in the motivation condition (though activation across subjects was correlated with emotion memory performance), and right OFC was correlated with performance in both conditions.

The pattern of activity in anterior insula cortex (AIC) was unlike that seen in other face network nodes. Bilateral AIC was activated in the baseline condition only, left AIC activity was not correlated with memory performance, but right AI was *negatively* correlated with memory performance in both runs (although right AIC activity did not reach significance at the group level in the motivated condition, peak values from each subject were correlated negatively with their emotion memory performance).

Finally, the amygdala exhibited a unique pattern of activity. While the left amygdala was equivalently active in both runs of the task, and performance on emotion memory was negatively correlated with left amygdala activation in the motivated run, the right amygdala was activated in the motivated condition only (see Figure 1).

As predicted, a midbrain region consistent with the location of the locus coeruleus (LC) was selectively activated during the motivated condition. Further, the hemodynamic response in this region was correlated with performance on memory for identity in both the baseline and motivated runs (though averaged groupwise activation in this region was not significant in the baseline condition, the amount of activity present across subjects was correlated with memory performance for that run).

### Amygdala: Condition by expression analysis

Since previous research has attributed right amygdala activity to threat processing, and the faces in memory task exhibited a mixture of expressions, a second analysis was conducted which analyzed responses separately for each combination of condition and expression: baseline and motivated viewing of anger, disgust, fear, happiness, sadness, and pain. The pattern of amygdala activity seen in the main analysis held within each expression category (see Table 2). Left amygdala responses were equivalent in baseline and motivated conditions, while right amygdala activations were absent in the baseline run and robust during the motivated condition. Further, threat-related expressions (fear and anger), elicited no greater amygdala activation than other expressions in either amygdala.

**Table 2:** Peak T-values for amygdala activation during face viewing, by expression class and condition.

		Left				Right			
		Peak T	x	y	z	Peak T	x	y	z
anger	baseline	3.11	−26	−2	−22	–	–	–	–
	motivated	2.97	−16	−8	−14	4.39	26	−6	−24
disgust	baseline	3.63	−22	−4	−14	–	–	–	–
	motivated	2.37	−26	0	−22	4.17	30	2	−24
fear	baseline	3.45	−22	0	−12	–	–	–	–
	motivated	3.8	−22	−2	−14	4.62	24	0	−24
happy	baseline	2.51	−22	−4	−24	–	–	–	–
	motivated	2.25	−20	−8	−16	7.51	30	−2	−28
sad	baseline	4.18	−22	−6	−14	–	–	–	–
	motivated	3.83	−16	−6	−14	6.19	22	−4	−26
pain	baseline	4.51	−22	−2	−14	–	–	–	–
	motivated	3.08	−22	0	−24	6.26	20	−6	−24

### Functional connectivity analyses

**Figure 3:**
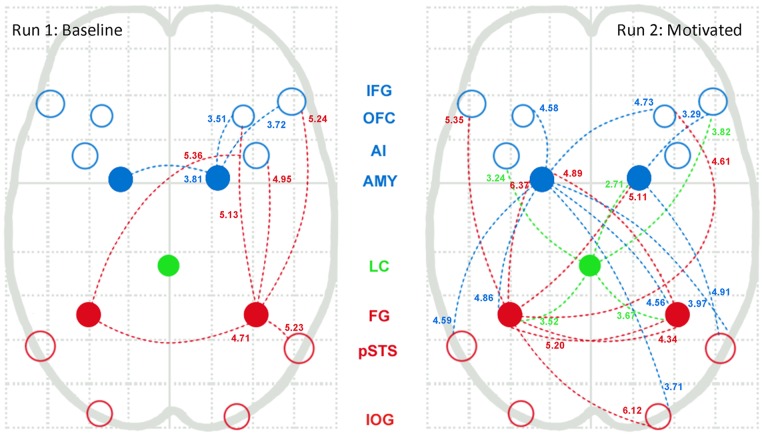
State-dependent changes in intra-network connectivity among face processing nodes. Results of the psychophysiological interaction (PPI) analysis among the nodes of the face-processing network. PPI seeds are displayed using filled circles. Other face network ROIs are empty circles. Nodes are labeled via the central column of abbreviations: core network nodes are displayed in red (FG  =  fusiform gyrus, IOG  =  inferior occipital gyrus, pSTS  =  posterior superior temporal sulcus), extended network nodes are displayed in blue (AMY  =  amygdala, AI  =  anterior insula, OFC  =  lateral orbitofrontal cortex, IFG  =  inferior frontal gyrus), and the locus coeruleus (LC) is in green. Numbers indicate the peak T value for the target region, and are positioned closer to the target on the line connecting the PPI seed node and the target node. Full results of the PPI analysis are available in Table S1.

Psychophysiological interaction analysis (PPI) was conducted using five regions as seeds. The fusiform gyrus and amygdala were selected bilaterally because of their putative deficits in disorders such as psychopathy and autism, and the locus coeruleus due to its control of ascending arousal associated with motivation. Changes in functional connectivity are consistent with a greater degree of functional integration both within the face-processing network and between these nodes and regions of the brain implicated in emotion processing, motivation, reward, and learning and memory, during the encoding of expressive faces. Results within the face-processing network are presented schematically in Figure 3, and full results are given in Table S1.

Three of the five seeds (left amygdala, left FG, and the LC) displayed significant connectivity with a greater extent of the face processing network during motivated processing of expressive faces. In the motivated condition, the left amygdala was significantly co-activated with the right IOG, bilateral FG, bilateral pSTS, and bilateral OFC. During baseline encoding it had significant connectivity only with a cluster of right amygdala. The left fusiform gyrus, similarly, displayed stronger functional connectivity with the face processing network during the motivated condition, with significant clusters in right IOG, right FG, bilateral amygdala, bilateral IFG, and right OFC. The locus coeruleus had no significant functional connectivity with the face processing network at baseline, but under motivation showed greater functional connectivity with bilateral FG, right IFG, left AIC, and right amygdala.

The final two seeds exhibited different patterns. The right amygdala had functional connectivity with the right IFG in both conditions, with the right OFC in the baseline condition only, and with right pSTS in the motivated condition only. The right fusiform had functional connectivity with complementary subsets of the network in each condition: with the right IFG, right AIC, right OFC, and right pSTS at baseline, and with the left amygdala and left FG during motivated face processing.

The five seed regions of interest showed additional functional connectivity during the motivated condition with regions associated with cognitive control, motivation, reward, arousal and memory. The dorsal anterior and anterior middle cingulate cortex exhibited functional connectivity during the motivated condition with bilateral FG, right amygdala, and the LC. The pre-supplementary motor area had significant PPI during the motivated condition with the bilateral FG and the left amygdala. The nucleus accumbens was significantly co-activated with the left FG and the right amygdala seeds: right amygdala was additionally co-activated with the ventromedial prefrontal cortex. The basal forebrain activity was significantlycorrelated with the right FG and LC seeds. PPI analyses did not show any significant effects with any of these regions in the baseline condition. All five seeds showed significant functional connectivity with medial temporal regions associated with memory consolidation (hippocampus and parahippocampal cortex) during the motivated run. In the baseline condition, only the right amygdala and the locus coeruleus were significantly covariant with these areas.

Regions that were co-activated with the PPI seeds during the baseline condition only are areas associated with emotion and theory of mind processing. Subgenual anterior cingulate cortex was coactive in the baseline run with right FG and the LC, temporal pole had significant baseline PPI with bilateral FG, left amygdala, and the LC, and the supramarginal and angular gyri had significant PPI with the bilateral FG.

## Discussion

The current study demonstrates that the manipulation of motivation to attend to emotionally expressive faceselicits selective changes both in the magnitude of responses in certain nodes of the face processing network and in the task-related functional connectivity among them.

The faces of human conspecifics are processed by an association of brain regions known as the face-processing network. Though this network has been well characterized over the past decade or more, the chief focus has been on the types of stimulus characteristics that best elicit activity in each region. Less understood are theinteractions between regions and effects of task demands on activity within and among nodes of the network, the complex interplay of mechanisms that make real-world dynamic face processing possible [41], [42]. Recent studies have begun use dynamic rather than static face stimuli, characterizing effects in BOLD response magnitude [43], and functional connectivity [44] to face stimuli of greater ecological validity.

New research highlights the idea that neural activity at the regional level is recruited not simply by the properties of the stimuli in the visual field, but by top-down factors like task demand and social context. In a task that involved systematic manipulations of stimulus properties and task demands, one study found that task demands, not stimulus characteristics, influence face processing regions [45]. For example, processing of face stimuli in the fusiform gyrus was enhanced not when face identity simply changed, but when changes in face identity were relevant for task performance. Additionally, a recent neuroimaging investigation documented selectively enhanced fusiform gyrus activation to faces of members of an arbitrary and temporary in-group, demonstrating that visual processing at the level of the core system of the face processing network is sensitive to top-down, short-term situational effects [46].

The current study investigated the effect of motivation on the neural processing of expressive faces during functional MRI. Participants watched short video clips of faces expressing happiness, sadness, fear, disgust, anger, and pain. After a surprise memory task, their motivation to attend to the faces was then manipulated by offering a small incentive to improve their memory performance on a second run of the task. During motivated perception, BOLD responses were greater in three regions associated with processing of emotion and of facial expression, the bilateral pSTS and the right amygdala. Activity in other nodes remained constant or decreased.

Additionally, the motivation manipulation affected the relationship between brain activity and behavior. Exclusively in the motivated condition, scores for emotion memory performance were significantly correlated with BOLD response magnitude for many face network nodes. Finally, greater coherence throughout the network in the motivated condition was measured with functional connectivity. Face network seed regions (bilateral fusiform gyrus and amygdala) had significant functional connectivity extensively and bilaterally during the motivated condition, while baseline connectivity was more sparse and right-lateralized.

Notably, increased motivation did not result in an across-the-board signal increase in the nodes of the face-processing network. Certain nodes (bilateral pSTS and right amygdala) exhibited greater activity in the second run, while others remained roughly the same or had slight numerical decreases in significance. This is likely due to the interplay of two offsetting factors – a specific and task-related increase in a subset of pertinent regions, working counter to a general habituation effect.

Due to the nature of the task design, it was necessary to follow a naïve baseline first run with a motivation manipulation and a motivated second run. For this reason, some changes between runs may be due to the effect of time on task or order. Although this complicates interpretation to some degree, the effects of habituation typically work in the opposite direction of the effects of interest. To our knowledge, no published investigation of face processing has ever demonstrated increases in BOLD response or in functional connectivity as a function of experience or time on task.

Habituation of the BOLD signal during face processing tasks is in fact a tool frequently used to identify which stimulus properties are tracked by a particular region.Known as fMRI-adaptation [47], or repetition suppression [48], the method has been employed in investigations of face processing (e.g., [49]–[51]). These effects are typically measured at the level of individual stimuli, but habituation effects can be observed during a second run of a face processing task even when a novel set of stimuli are used in each run. In one study, Kleinhansand co-authors [14] measured habituation to face stimuli by comparing activation to all faces in a second run of the task to that in the first and found significant reductions in signal in the amygdala and fusiform gyrus. Their paradigm differed from traditional repetition suppression task designs by using a different set of photographs of faces for each run, and thus demonstrate a more global task-level habituation effect. Thus, in the current task, signal increases seen in pSTS and right amygdala during the second run of the task cannot be due to experience or time on task, as these would produce decreases rather than increases in signal.A more likely interpretation is that in the second run of our task, information about the emotion expression became more salient due to task instructions. As such, regions associated with emotion and expression processing (amygdala and pSTS) were recruited to a greater degree.

Similarly, we observed increased functional connectivity during motivated viewing of faces. Though habituation effects have not been thoroughly investigated in PPI, evidence exists that functional connectivity during face processing also undergoes habituation over time. A recent dynamic causal modeling (DCM) study of face processing [52] demonstrated in a multi-phase design that connectivity between the fusiform gyrus and the amygdala decreased within subjects at the second presentation of previously-seen face stimuli, when the faces were less salient than their initial exposure. Interestingly, the DCM analysis could only be conducted on right-side face processing nodes at time two, because left side clusters were not of sufficient significance to serve as nodes in the model. Thus, evidence suggests that changes due to time on task would not produce the changes observed in the current study, and the more extensive functional connectivity seen here is due to the manipulation of motivation to attend to the stimuli.

### Correlations with behavior

The neuro-hemodynamic activity in the face processing network during baseline viewing of dynamic expressive faces was not correlated with subsequent performance on the memory test for identity and expression, with the exception of the left IOG and right OFC, which were positively correlated with emotion memory performance, and the right anterior insula, which was negatively correlated with memory for identity. In the motivated condition, the magnitude of activity was closely tied to memory for emotional expression, reaching significance in all face processing nodes except for the left amygdala and the right anterior insula, both of which were negatively correlated with memory for identity.The AIC is polysensory cortex involved in mapping internal states of bodily and subjective feeling, and plays a crucial role in emotional awareness, as well as facilitating the detectionof important environmental stimuli [53].

### Functional connectivity

PPI analysis revealed more cohesive function among nodes of the face network, and provided evidence of the integration of these nodes with other regions involved in emotion processing, motivation, reward, and learning and memory. The analyses were conducted originally using bilateral fusiform gyrus and bilateral amygdala seeds due to their prominent role both in face processing and in investigations of neuropsychiatric disorders. The locus coeruleus was also investigated as a seed region because of its unique and broad contribution to motivation and affective processing (see Table S1).

Bidirectional functional connectivity was present between the left amygdala and bilateral fusiform gyrus, but this was seen exclusively during the motivated condition. This finding is of crucial significance. A study investigated functional connectivity during face processing in individuals with autism spectrum disorder and controls, finding reduced connectivity between the left amygdala and the fusiform gyrus in ASD patients, and quite interestingly, a reverse correlation between the amygdala-FG connectivity and clinical ratings of social symptom severity [54]. The authors concluded that abnormal connectivity between these regions may contribute to social impairments in ASD. The current findings, however, imply the reverse of this causal relationship, that differences in interest in the task or motivation to attend to the stimuli can be reflected in changes in functional connectivity between these very same regions.

Under augmented motivation, the LC was robustly activated at the group level and strongly correlated with memory performance. Further, exclusively in the motivated condition the LC exhibited functional connectivity with many regions of the face processing network (bilateral IOG, bilateral FG, right IFG, bilateral OFC, and bilateral amygdala), as well as the basal forebrain, insula, medial prefrontal cortex, anterior midcingulate cortex, and dorsal ACC.

During the motivated condition, several regions known to be involved in functions that are relevant to the motivated processing of expressive faces appeared as clusters having significant PPI with multiple seed regions. These include the temporal poles, hippocampus and parahippocampal cortex, anterior paracingulate cortex, anterior midcingulate cortex, posterior cingulate cortex, the basal forebrain, and the pre-supplementary motor area.

The anterior paracingulate cortex and the posterior cingulate cortex are involved in the process by which unfamiliar faces become familiar, identified in tasks which used neutral face stimuli [55] and emotional face stimuli [56], respectively.Also, the pre-supplementary motor area is best known for action preparation and observation [57], but it also processes facial expression [58]–[60] and judgments of facial familiarity [55], [61]. These regions, as well as the hippocampus and parahippocampal cortex, were recruited to assist in memory formation for faces during the second run of the task.

Additionally, regions associated with motivation and goal-directed attention displayed functional connectivity with multiple nodes of the face processing network under motivation. The anterior midcingulate cortex is known to assist in the maintenance of goal-directed attention [62], [63]. The basal forebrain is a collection of nuclei that receive input from the central nucleus of the amygdala and project cholinergic afferents to widespread cortical regions, which is believed to mediate amygdala influence on sensory processing in the cortex [64], and has been shown to track motivation and effort [65].

### Amygdala laterality

A surprising pattern of response was detected in the amygdala. While the left amygdala was equally active in both runs of the task and correlated negatively with task performance in the motivated condition, the right amygdala was selectively active at the group level only in the motivated condition, and was positively correlated with memory performance. Further, this pattern was observed individually within each of the expression categories. To address whether spurious response patterns among the six emotions could explain these results, an ancillary analysis was conducted in which faces were classified by both motivational condition and expression identity. Even with the reduced power to detect signal change, the same pattern was observed within each of the six emotional expressions. The left amygdala activation was significant in both conditions and roughly insensitive to motivation manipulation, and right amygdala activation was absent during baseline and robust during the motivated condition (Table 2, Figure 1).

In other words, the current study revealed a positive effect of motivation in the right amygdala, and responses in the left amygdala that were significant, consistent, and insensitive to motivational state. Though motivational relevance is an effect of laterality that to our knowledge has not yet been discussed in the literature, it is consistent with the extant literature. A meta-analysis of amygdala laterality [66] found no laterality differences according to “task demand,” but this was operationalized as implicit versus explicit tasks, a concept that is orthogonal to motivation.

A number of imaging studies which report amygdala responses to emotionally-valenced or face stimuli, particularly those in which attention is diverted to a “distractor” task, have found activation in the left amygdala only [5], [67]–[69]. When Vuilleumier and colleagues [31] reported equivalent amygdala activation to attended and unattended fearful faces, lending support to the notion that amygdala responses are “automatic,” the effect was only seen in the left amygdala. Similarly, neural responses to fear faces which resisted suppression in a binocular rivalry task were again found in the left amygdala only [32]. This is consistent with the current findings, in which a positive effect of motivation was seen in the right amygdala, but significant responses in the left amygdala were insensitive to motivational state.

Several studies have elaborated on this lateralized effect, finding that left amygdala responses to repeated presentations of emotional stimuli remain constant while right amygdala responses quickly habituate [70]–[72]. The involvement of right amygdala in response to salient or motivationally relevant stimuli is also consistent with right-dominated amygdala involvement in fear conditioning [73], [74], and the observation of physiological hypoarousal in right amygdala lesion patients [75], [76]. In an imaging paradigm of aversive conditioning using angry faces as stimuli [77], the left amygdala responded equivalently to angry faces across presentation conditions, while the right amygdala responded selectively to stimuli of motivational significance (angry faces that had been paired with an aversive white noise burst).

Thus, the literature supports a consistent and indiscriminate (more automatic) response to emotional content in the left amygdala, and greater sensitivity to motivational significance in the right. The implications of this putative task-dependent laterality effect are non-trivial. For instance, a recent study investigated amygdala responses to emotional faces across different task demands, using a functional localizer to identify face-responsive amygdala voxels [78]. All conclusions about amygdala function in the paper were drawn from this ROI, which was defined using passive viewing trials and thus identified a cluster in the left amygdala only. If the right amygdala contributed to the other, evaluative conditions, perhaps in a pattern different from that seen in left amygdala, those potential results were discarded from the analysis due to the task demands of the passive functional localizer.

### Conclusions

In healthy individuals, an augmented motivation to attend to and process emotionally expressive faces results in widespread changes in the regions and networks involved face processing.This externally-imposed change in motivational context could parallel the natural variation that is brought into the scanning suite via individual differences between participants. Under certain conditions these differences are of no consequence and are harmlessly swept into the statistical model with all other sources of noise. If however, the motivation to attend to stimuli and participate in the task covaries with group membership, as it might in neuropsychiatric populations such as psychopathy [29] and autism [16], [17], special care must be taken to attempt to disentangle the effects of motivation and their neural sequelae from true pathology in the relevant circuits supporting the behavior.

## Supporting Information

Table S1 Full results of connectivity (PPI) analysis by seed and by run.
(PDF)
