# Supplementary material for: Passive and Motivated Perception of Emotional Faces: Qualitative and Quantitative Changes in the Face Processing Network
Source: PLoS One. 2012 Jun 29;7(6):e40371. doi: 10.1371/journal.pone.0040371 (PMC3386961; doi:10.1371/journal.pone.0040371)
Supplement: Table S1 — Full results of connectivity (PPI) analysis by seed and by run. (PDF) [file pone.0040371.s001.pdf]

Table S1: Full results of connectivity (PPI) analysis by seed and by run.

A. LEFT FUSIFORM SEED											
**RUN 1 - BASELINE**						**RUN 2 - MOTIVATED**					
Area		Peak T	x	y	z	Area		Peak T	x	y	z
R	**fusiform gyrus**	4.71	38	-56	-14	R	**inferior occipital gyrus**	6.12	30	-96	-4
R	**antierior insula**	5.36	36	14	8	R	**fusiform gyrus**	4.34	26	-58	-16
						R	**amygdala**	6.37	26	4	-18
R	**subgenual anterior cingulate cortex**	3.67	2	26	-4	L	**amygdala**	5.11	-22	0	-14
R	**supramarginal gyrus**	4.3	68	-32	24	L	**inferior frontal gyrus (triangular part)**	5.35	-38	30	6
R	**angular gyrus**	4.26	44	-62	48	L	**inferior frontal gyrus (triangular part)**	5.18	-44	42	10
R	superior frontal gyrus	5.97	28	50	8	R	**lateral orbitofrontal cortex**	4.61	48	30	-18
L	middle frontal gyrus	5.1	-32	34	16						
R	middle frontal gyrus	4.91	44	52	8	R	**temporal pole**	4.92	56	10	-18
R	paracentral lobule	3.4	6	-18	48	R	**pre-supplementary motor area**	4.39	8	10	68
R	superior temporal gyrus	4.59	66	-30	2	L	**pre-supplementary motor area**	4.56	-6	12	70
L	superior temporal gyrus	4.21	-48	-18	-4	R	**nucleus accumbens**	3.98	4	6	-10
R	middle temporal gyrus	4.2	68	-38	-4	L	**nucleus accumbens**	4.64	-6	4	-10
L	middle temporal gyrus	3.83	-62	-38	-2	R	**basal forebrain**	5.59	8	6	-18
R	precuneus	4.9	8	-54	56	R	**hippocampus**	7.42	22	-20	-12
R	superior parietal lobule	3.73	18	-62	58	R	superior medial prefrontal cortex	4.95	2	52	20
R	thalamus	4.32	22	-20	8	R	**dorsal anterior cingulate cortex**	5.11	2	30	22
L	caudate	3.98	-16	14	14	R	**anterior middle cingulate cortex**	5.3	4	-6	42
						R	superior frontal gyrus	4.76	24	46	18
						L	superior frontal gyrus	4.42	-18	48	18
						L	superior temporal gyrus	4.33	-60	-24	12
						L	cuneus	4.62	0	-84	20
						R	lingual gyrus	7.59	12	-54	-6
						L	lingual gyrus	5.02	-10	-54	-2
						R	middle occipital gyrus	5.01	36	-76	22
						L	middle occipital gyrus	5.24	-28	-74	20

B. RIGHT FUSIFORM SEED

RUN 1 - BASELINE						RUN 2 - MOTIVATED					
Area		Peak T	x	y	z	Area		Peak T	x	y	z
R	**inferior frontral gyrus (triangular part)**	5.24	50	34	16	L	**fusiform gyrus**	5.2	-36	-58	-18
R	**anterior insula**	4.95	34	14	10	L	**amygdala**	4.89	-22	-6	-10
R	**lateral orbitofrontal cortex**	5.13	36	62	-2						
R	**posterior superior temporal gyrus**	5.23	64	-32	4	R	**temporal pole**	3.35	28	18	-28
						L	**temporal pole**	3.36	-44	24	-16
R	**supramarginal gyrus**	4.83	68	-32	28	R	**pre-supplementary motor area**	4.78	6	10	72
R	**angular gyrus**	5.03	48	-60	44	L	**pre-supplementary motor area**	4.36	-4	10	70
R	superior frontal gyrus	6.34	26	50	8	R	**basal forebrain**	5.18	14	-6	-12
R	middle frontal gyrus	4.83	42	50	6	R	**hippocampus**	3.87	40	-22	-16
L	middle frontal gyrus	5.7	-26	56	12	L	**hippocampus**	3.88	-36	-18	-18
L	paracentral lobule	5.66	0	-26	50	L	**anterior middle cingulate cortex**	3.52	0	-8	42
L	middle temporal gyrus	4.91	-64	-24	2	L	superior medial prefrontal cortex	3.89	-8	54	18
R	middle temporal gyrus	4.89	54	-68	18	R	superior frontal gyrus	3.75	26	48	14
L	inferior parietal lobule	5.38	-40	-52	36						
R	precuneus	5.28	10	-52	54						
L	middle occipital gyrus	5.85	-48	-70	4						
R	calcarine	5.36	22	-90	0						
R	medial dorsal thalamus	4.82	6	-12	6						
L	putamen	5.03	-20	8	6						
L	caudate nucleus	4.51	-10	18	6						

C. LEFT AMYGDALA SEED

RUN 1 - BASELINE

Area		Peak T	x	y	z
R	**amygdala**	3.81	28	2	-24
L	**medial orbitofrontal cortex**	3.29	-6	50	-8
R	cuneus	4.55	12	-88	20
R	lingual gyrus	3.86	24	-66	-4
L	calcarine	3.47	-10	-78	10

RUN 2 - MOTIVATED

Area		Peak T	x	y	z
R	**inferior occipital gyrus**	3.71	26	-98	-6
R	**fusiform gyrus**	4.56	36	-54	-12
L	**fusiform gyrus**	4.59	-36	-64	-16
R	**posterior superior temporal sulcus**	3.97	50	-76	16
L	**posterior superior temporal sulcus**	4.86	-52	-70	24
R	**lateral orbitofrontal cortex**	4.73	34	26	-24
L	**lateral orbitofrontal cortex**	4.58	-22	12	-24
L	**temporal pole**	5.94	-34	22	-24
R	**pre-supplementary motor area**	4.56	-4	14	44
L	**anterior hippocampus**	3.88	-30	-10	-14
L	**posterior hippocampus**	4.43	-36	-42	-4
R	**parahippocampal cortex**	4.5	36	-34	-14
L	superior medial prefrontal cortex	4.62	-2	-52	40
L	posterior cingulate gyrus	4.41	-2	-46	32
L	middle frontal gyrus	4.69	-26	42	34
L	precentral gyrus	6.61	-32	0	44
R	middle temporal gyrus	4.52	44	-74	26
L	middle temporal gyrus	6.61	-30	-22	-16
R	posterior inferior temporal gyrus	3.91	52	-64	-8
L	inferior temporal gyrus	4.63	-60	-24	-16
L	lingual gyrus	4.01	-14	-38	-6
L	hypothalamus	4.04	-8	-2	-6
L	substantia nigra	4.02	-14	-22	-8
R	caudate body	3.77	18	-14	20
L	caudate body	4.29	-12	0	20
R	superior colliculus	4.26	4	-22	-4
L	superior colliculus	3.66	-4	-24	-4

D. RIGHT AMYGDALA SEED											
**RUN 1 - BASELINE**						**RUN 2 - MOTIVATED**					
	Area	Peak T	x	y	z		Area	Peak T	x	y	z
R	**inferior frontal gyrus (triangular part)**	3.72	54	24	26	R	**inferior frontal gyrus (triangular part)**	4.91	48	36	-4
R	**lateral orbitofrontal cortex**	3.51	34	34	-22	R	**posterior superior temporal sulcus**	3.29	50	-46	16
L	**temporal pole**	3.78	-44	16	-28	R	**nucleus accumbens**	3.83	8	10	-6
R	**parahippocampal cortex**	4.9	24	6	-26	L	**nucleus accumbens**	5.36	-10	6	-8
L	**hippocampus**	3.69	-26	-6	-22	R	**basal forebrain**	4.04	12	6	-14
R	middle frontal gyrus	3.88	48	42	10	R	**medial orbitofrontal cortex**	3.7	2	54	-6
R	supramarginal gyrus	3.47	42	-32	40	R	**parahippocampal cortex**	5.3	30	-2	-34
R	middle temporal gyrus (MT)	4.7	52	-66	-2	R	**hippocampus**	3.66	30	-8	-26
L	middle temporal gyrus (MT)	3.84	-66	-40	-2	L	**hippocampus**	3.14	-22	-26	-14
R	middle occipital gyrus	4.2	42	-82	10	R	**dorsal anterior cingulate cortex**	3.51	2	38	12
L	middle occipital gyrus	4.07	-44	-88	10	R	superior medial prefrontal cortex	3.25	8	52	18
L	thalamus	4.39	-12	-22	-2	R	superior temporal gyrus	3.63	52	-26	-4
						R	middle temporal gyrus (MT)	4.32	54	-72	6
						L	middle temporal gyrus	3.61	-52	-22	-12
						L	lingual gyrus	3.18	-12	-54	0
						R	thalamus	4.24	8	-6	-2
						L	thalamus	4.53	-8	-24	0

E. LOCUS COERULEUS SEED

RUN 1 - BASELINE

	Area	Peak T	x	y	z
R	**parahippocampal cortex**	3.12	26	-24	-22
L	**subgenual anterior cingulate cortex**	3.27	-2	18	-6
R	precentral gyrus	3.27	44	0	34
L	superior temporal gyrus	3.74	-52	-26	0
R	middle temporal gyrus	3.51	66	-12	-4
R	lingual gyrus	3.18	10	-46	4
L	middle occipital gyrus	3.61	-26	-82	14
L	middle occipital gyrus	3.11	-42	-78	14
	midbrain	3.77	2	-36	-14

RUN 2 - MOTIVATED

	Area	Peak T	x	y	z
R	**fusiform gyrus**	3.67	36	-64	-20
L	**fusiform gyrus**	3.52	-36	-34	-24
R	**inferior frontal gyrus (triangular)**	3.82	44	12	24
L	**anterior insula**	3.24	-28	14	-14
R	**amygdala**	2.71	22	4	-16
R	**temporal pole**	3.67	34	26	-26
R	**basal forebrain**	3.73	10	0	-14
R	**parahippocampal cortex**	4.18	36	-18	-26
L	**hippocampus**	3.54	-26	-10	-22
	**dorsal anterior cingulate cortex**	3.47	0	32	14
	**anterior midcingulate cortex**	3.78	0	6	32
R	superior medial prefrontal cortex	3.51	8	52	24
L	medial prefrontal cortex	3.04	-4	58	0
L	posterior cingulate cortex	3.21	-2	-44	32
R	precuneus	3.64	10	-78	56
L	superior parietal lobule	3.73	-32	-62	62
L	calcarine	5.02	-4	-84	-12
L	lingual gyrus	4.42	-30	-84	-14
	midbrain	4.86	0	-36	-16
